# Allostatic load profiles, resilience, and psychosocial adaptation in young adults

**DOI:** 10.3389/fpsyg.2026.1860265

**Published:** 2026-07-30

**Authors:** Se Jun Koo, Jung Woo Park, Jee Eun Min, Eun Lee, Suk Kyoon An

**Affiliations:** 1 Section of Self, Affect and Neuroscience, Institute of Behavioural Science in Medicine, Yonsei University College of Medicine, Seoul, Republic of Korea; 2 Department of Psychiatry, Yonsei University College of Medicine, Severance Hospital, Seoul, Republic of Korea; 3 Graduate Program in Cognitive Science, Yonsei University, Seoul, Republic of Korea

**Keywords:** allostatic load, depression, latent profile analysis, psychosocial functioning, resilience, young adults

## Abstract

**Introduction:**

Allostatic load (AL) reflects the cumulative physiological burden on the body, quantified using biomarkers across multiple systems. The current study identified latent AL profiles in young adults and examined whether resilience moderates associations between AL and depressive symptoms, social functioning, and role functioning.

**Methods:**

A total of 165 nonclinical young adults (84 women; aged 19–30 years) provided data on 15 biomarkers spanning the hypothalamic-pituitary-adrenal axis, oxidative stress, inflammatory-immune, lipid/glucose-metabolic, and renal systems, which were z-standardized. Latent profile analysis was conducted using the *mclust* package in R. Moderated regression models examined depressive symptoms, social functioning, and role functioning as outcomes of AL profile, resilience, and their interaction, adjusting for age, sex, and cognitive ability. Percentile bootstrap confidence intervals were estimated (*R* = 5,000).

**Results:**

A two-profile solution was retained based on classification quality and interpretability, yielding control and higher-dysregulation profiles (*n* = 132 and *n* = 33, respectively; entropy = 0.965; average posterior probability = 0.993). The higher-dysregulation profile showed relatively elevated dysregulation, particularly in inflammatory-immune and lipid/glucose-metabolic markers. Resilience was inversely associated with depressive symptoms [*b* = −0.138, *p* < 0.001, 95% CI (−0.199, −0.079)], and a significant AL profile × resilience interaction [*b* = −0.335, *p* < 0.001, 95% CI (−0.645, −0.055)] indicated that this association was stronger in the higher-dysregulation group. A similar interaction was observed for social functioning (*b* = 0.038, *p* < 0.001, 95% CI [0.003, 0.063]), whereas the interaction for role functioning was not significant.

**Discussion:**

These findings suggest that person-centered AL profiles capture heterogeneous physiological risk patterns. Resilience showed stronger associations with lower depressive symptoms and better social functioning among individuals with relatively greater physiological dysregulation, highlighting profile-dependent links between psychological resilience and psychosocial adaptation in young adulthood.

## 1 Introduction

Allostasis refers to a regulatory process in which physiological systems, including the hypothalamic-pituitary-adrenal (HPA) axis, inflammatory-immune responses, and metabolic regulation, are mobilized to maintain stability in response to stress (Sterling and Eyer, 1988). When these systems are repeatedly or chronically activated, the body may experience cumulative physiological “wear and tear”, referred to as allostatic load (AL) (McEwen and Stellar, 1993). AL was originally conceptualized to capture the physiological cost of chronic stress and is typically quantified by aggregating biomarkers across multiple systems, including the HPA axis (e.g., cortisol), oxidative stress (e.g., homocysteine), inflammatory-immune (e.g., C-reactive protein), lipid metabolism (e.g., total cholesterol), glucose metabolism (e.g., insulin), and renal function (e.g., cystatin C) (Seeman et al., 2001; Juster et al., 2010; McCrory et al., 2023).

Previous studies have linked AL to a wide range of adverse outcomes, including depressive symptoms, cognitive decline, and impaired psychosocial functioning (Guidi et al., 2020; Lenart-Bugla et al., 2022; Pfaltz and Schnyder, 2023). Large population-based cohorts (e.g., UK Biobank; the English Longitudinal Study of Ageing) consistently show that higher AL is associated with greater depressive symptomology (de Oliveira et al., 2023; Gou et al., 2025). For example, among older adults, a one-unit increase in AL has been associated with approximately a 1.21-point increase in Center for Epidemiologic Studies Depression Scale (CES-D) scores, which is comparable in magnitude to antidepressant use (Kobrosly et al., 2014). Similarly, analyses by diagnostic status indicate that, after adjusting for demographic and lifestyle factors, high AL ( ≥ 4) is associated with increased odds of major depressive disorder [MDD; odds ratio (OR) = 2.27–2.96] (Honkalampi et al., 2021).

Beyond mental health, AL is closely linked to functioning in social and role domains. Positive social relationships, including supportive interactions and reliable assistance (e.g., caring, listening, and reliable help) are associated with lower AL, whereas relationship strain and negative interactions (e.g., criticism, excessive demands, and feeling let down) are linked to higher AL (Seeman et al., 2002; Seeman et al., 2004; Brooks et al., 2014). Longitudinal evidence indicates that elevated AL predicts subsequent limitations in activities of daily living and instrumental activities of daily living (Zhao et al., 2024). From a life-course perspective, social engagement and AL may operate in parallel pathways linking early adversity to adult multimorbidity, positioning AL as an integrative biomarker of both biological and social processes (Atkinson et al., 2023). Together, these findings suggest that AL may have a key role in shaping both social functioning and everyday role performance.

Young adulthood (ages 18–29 years) represents a critical developmental period characterized by increasing autonomy, identity formation, and the assumption of social roles, all of which may heighten exposure to stressors (Arnett et al., 2014). Concurrently, this period is marked by substantial neurobiological plasticity. Stress-related neural processes remain modifiable, and some stress-induced brain changes appear reversible. For instance, frontoparietal connectivity and attentional control have been shown to recover following stress reduction within individuals (Liston et al., 2009). This broader plasticity supports the view that stress-related neurobiological changes may be mitigated through reduced stress exposure or targeted interventions (McEwen, 2012). Consistent with this, early preventive interventions have been shown to produce sustained reductions in inflammatory markers into young adulthood (Miller et al., 2014). Collectively, these findings highlight young adulthood as a key window for identifying and modifying AL-related risk.

Resilience is widely discussed in health psychology as a construct relevant to mental health and adaptation under stress. In the present study, resilience is conceptualized as the capacity to maintain or regain psychological wellbeing and adaptive functioning in the context of stress or adversity. Importantly, resilience can be understood either as a dynamic adaptive process that unfolds over time or as a relatively stable, self-reported psychological trait or resource. Because the present study used a cross-sectional design and a self-report resilience scale, resilience was operationalized as measured psychological resilience rather than as a directly observed longitudinal recovery process. Clinical studies in populations such as individuals with schizophrenia spectrum disorders and cancer have linked higher resilience to better quality of life and psychosocial functioning (Wambua et al., 2020; Zhao et al., 2022). Meta-analytic and review evidence similarly demonstrates inverse associations between resilience and depression, as well as other adverse mental health outcomes (Hu et al., 2015; Wermelinger, Ávila et al., 2017; Färber and Rosendahl, 2018). Beyond mental health, resilience may also offer protection against physiological dysregulation. For example, a stronger sense of purpose has been associated with lower subsequent AL at long-term follow-up (Zilioli et al., 2015), and broader psychosocial resources (e.g., mastery and social support) show similar inverse relationships (Wiley et al., 2017). However, resilience does not uniformly correspond to lower biological risk. Longitudinal research in rural African American youth has demonstrated that individuals exhibiting high psychosocial competence under adversity may nonetheless show elevated AL in early adulthood, a phenomenon termed “skin-deep resilience” (Brody et al., 2013). Similarly, in low socioeconomic contexts, greater self-control has been associated with better psychosocial outcomes but accelerated epigenetic aging (Miller et al., 2015). Overall, these findings highlight the importance of examining how resilience shapes the relationship between AL and psychosocial outcomes.

Traditional approaches to measuring AL typically involve dichotomising biomarkers using distribution-based thresholds (e.g., highest-risk quartiles) and summing the number of high-risk indicators (Seeman et al., 2001). Although this approach facilitates comparison across studies, it assumes equal weighting of biomarkers and relies on sample-specific cutoffs. Moreover, additive indices may obscure heterogeneity in physiological profiles. These limitations have been widely noted in methodological reviews and consensus statements (Juster et al., 2010; McLoughlin et al., 2020; McCrory et al., 2023). In contrast, person-centered approaches such as latent profile analysis (LPA) allow for the identification of subgroups with distinct biomarker patterns across physiological systems (Masyn, 2013; Nylund-Gibson and Choi, 2018). These profiles may describe different patterns of dysregulation and may be associated with variation in psychological and functional outcomes. Recent studies have used LPA to identify profiles such as low-risk, inflammatory, and cardiovascular patterns, linking these to mental health outcomes (Carbone, 2021), social factors (Johnson et al., 2019), and other health outcomes, including chronic pain (Liang and Booker, 2024).

Guided by this perspective, the present study focused on young adults and aimed to (1) derive latent AL profiles from multisystem biomarkers, (2) examine associations between these profiles and depressive symptoms, and (3) test whether resilience moderates these relationships. This research question is important because young adulthood represents a developmental period in which stress-related physiological dysregulation may begin to emerge before more established health problems become clinically manifest later in adulthood, while psychological and social functioning remain potentially modifiable. From a health psychology perspective, examining AL profiles together with resilience may help clarify why individuals with similar levels of physiological dysregulation differ in emotional and functional adaptation. By situating AL within a developmental period marked by both heightened stress exposure and ongoing neurobiological plasticity, this study aimed to clarify whether AL functions as a marker of relative physiological dysregulation associated with psychosocial adaptation in early adulthood.

## 2 Materials and methods

### 2.1 Participants

A total of 166 young adults were recruited through online advertisements. One participant was excluded due to missing cortisol data resulting from sample contamination, yielding a final sample of 165 participants (84 females and 81 males; mean age = 23.05 ± 2.59 years; range = 19–30 years). Participants were screened for current and past psychiatric and neurological conditions using the Mini-International Neuropsychiatric Interview (MINI) (Sheehan et al., 1998; Yoo et al., 2006). The study was approved by the Severance Hospital Research Review Board (IRB Nos. 4-2014-0744 and 2014-1767-035), and all participants provided written informed consent. All procedures followed institutional guidelines and the Declaration of Helsinki.

### 2.2 Measures

#### 2.2.1 Beck depression inventory

Depressive symptoms were assessed using the 21-item Beck Depression Inventory (BDI) (Beck et al., 1988; Rhee et al., 1995). Items are rated on a 4-point scale (0–3), yielding a total score ranging from 0 to 63, with higher scores indicating greater symptom severity. Internal consistency was high in the present sample (Cronbach’s α = 0.89).

#### 2.2.2 Global functioning: social and role scales

Psychosocial functioning was assessed using the Global Functioning: Social (GF:S) and Global Functioning: Role (GF:R) scales (Cornblatt et al., 2007). The GF:S evaluates the quality and quantity of age-appropriate social relationships, whereas the GF:R assesses role performance in domains such as education, employment, and daily responsibilities, relative to developmental expectations. These clinician-rated measures were originally developed for youth at clinical high risk for psychosis but have since been applied to broader populations (Lucarini et al., 2024).

Ratings were assigned by a trained psychologist based on clinical interview and standardized anchor descriptions. Each scale yields scores ranging from 1 (extreme dysfunction) to 10 (superior function). Although multiple indices (current, lowest, highest past year functioning) are available, the present study used current functioning (past month). GF:S and GF:R ratings were completed before biomarker-based AL profiles were derived, and the rater did not have access to participants’ biomarker profile classifications at the time of assessment.

#### 2.2.3 Connor-Davidson resilience scale

Psychological resilience was measured using the 25-item Connor-Davidson Resilience Scale (CD-RISC) (Connor and Davidson, 2003; Baek et al., 2010). The CD-RISC assesses self-reported psychological resilience, including perceived capacity to cope with stress, adapt to change, maintain goal-directed effort, and recover from adversity. In the present cross-sectional study, CD-RISC scores were interpreted as a measured psychological trait/resource rather than as evidence of a longitudinal adaptive process. Items are rated on a 5-point scale (0–4), with total scores ranging from 0 to 100; higher scores indicate greater resilience. Internal consistency was excellent (Cronbach’s α = 0.93).

#### 2.2.4 Standard progressive matrices

Cognitive ability was measured using the Standard Progressive Matrices (SPM) (Raven et al., 1990), a nonverbal reasoning test consisting of 60 matrix problems. Scores reflect the number of correct responses (range: 0–60). The SPM has demonstrated strong reliability and cross-cultural validity (Raven, 2000).

### 2.3 Biomarker collection and processing

Blood samples were collected via venipuncture during one of two broad collection windows, either in the morning (09:00–11:00) or in the afternoon (13:00–15:00). Samples were centrifuged, aliquoted into microtubes, and stored at −80°C before analysis. Samples were analyzed at Seoul Clinical Laboratories using validated immunoassays and automated clinical chemistry analysers under standard quality control procedures. Participants were screened for current and past psychiatric and neurological conditions as part of the eligibility assessment. However, detailed biomarker-specific pre-collection information, including exact blood draw timing within each collection window, fasting status, recent infection or acute inflammatory symptoms, medication use at the time of blood collection, acute exercise, and sleep before blood collection, was not systematically available in a form that allowed statistical adjustment or biomarker-specific exclusion. All biomarkers were z-standardized prior to analysis.

### 2.4 Statistical analysis

All statistical analyses were conducted using R (version 4.4.1). LPA was performed using the *mclust* package (Scrucca et al., 2016) to identify subgroups based on physiological dysregulation. Fifteen serum biomarkers were included, spanning multiple systems: HPA-axis [cortisol, dehydroepiandrosterone sulfate (DHEA-S)], oxidative stress (uric acid, homocysteine), inflammatory-immune [high-sensitivity C-reactive protein (hs-CRP), albumin, interleukin-6 (IL-6)], lipid metabolism [total cholesterol, HDL cholesterol (HDL-C), triglycerides (TG)], glucose metabolism (glucose, insulin), and renal function [blood urea nitrogen (BUN), creatinine, cystatin C].

Model selection was guided by fit indices [Bayesian Information Criterion (BIC) (Schwarz, 1978), Bootstrap Likelihood Ratio Test (BLRT) (McLachlan and Peel, 2000)], classification indices [entropy (Celeux and Soromenho, 1996), average posterior probabilities (AvePP)(Masyn, 2013)], and stability metrics [Adjusted Rand Index (ARI) (Hubert and Arabie, 1985)]. Profile enumeration was based on a multi-criteria approach rather than a single fit index. BIC and BLRT were considered as statistical fit indices, whereas entropy and AvePP were used to evaluate classification certainty and profile separation. Profile size, substantive interpretability, parsimony, and resampling-based internal validation were also considered when selecting the retained solution. This approach is consistent with methodological guidance recommending that class enumeration be evaluated using multiple sources of evidence, including statistical fit, classification quality, substantive interpretability, and parsimony (Nylund et al., 2007; Masyn, 2013; Nylund-Gibson and Choi, 2018). Because class enumeration can be influenced by sample size, number of indicators, class separation, and model complexity, more complex solutions were interpreted cautiously in the present sample (Tein et al., 2013). The resulting AL profile variable was treated as a categorical predictor in moderated regression models examining depressive symptoms (BDI), social functioning (GF:S), and role functioning (GF:R). CD-RISC was mean-centered, and AL profile was dummy-coded. All models were adjusted for age, sex, and cognitive ability (SPM).

Missing data were minimal. Two CD-RISC items (two participants) and five BDI items (four participants) were imputed using person-level mean substitution to permit complete score calculation. All regression models were percentile bootstrapped (5,000) (Efron and Tibshirani, 1993). Model assumptions were evaluated using variance inflation factors ( < 2) for multicollinearity (Belsley et al., 1980; O’Brien, 2007; Zuur et al., 2010) and Cook’s distance ( > 0.5) to identify influential cases (Cook, 1977). Significant interactions were investigated using simple slopes analyses using the *sim_slopes()* function from the *interactions* package (Long, 2024). All tests were two-tailed with a significance threshold of *p* < 0.05.

## 3 Results

### 3.1 Latent profile analysis of allostatic load

LPA was conducted using 15 z-standardized AL biomarkers. Fit indices for the examined profile solutions are presented in Table 1. Based on the BIC, the five-profile solution showed the best fit (BIC = -6010.53; higher values indicate better fit in *mclust*). However, the retained solution was selected using the multi-criteria approach described above rather than BIC alone. The BLRT supported the addition of a second profile (*k* = 1 vs. *k* = 2, *p* = 0.002), but not a third (*k* = 2 vs. *k* = 3, *p* = 0.513). The BLRT was also significant for the four-profile solution (*k* = 3 vs. *k* = 4, *p* = 0.002), indicating that the BLRT criterion alone did not uniquely support the two-profile solution. However, the two-profile solution showed the highest entropy (0.965) and highest AvePP (0.993), indicating strong classification certainty. Consistent with this, profile-specific average posterior probabilities were high for both the control profile (0.996) and the higher-dysregulation profile (0.983). Among the full-sample fit indices, the ARI was also highest for the two-profile solution (ARI = 0.429).

**TABLE 1:** Latent profile analysis fit indices (*N* = 165).

Number of profiles	BIC	Entropy	AvePP	ARI	BLRT, *p* (*k* vs. *k*-1)
2	−6272.01	0.965	0.993	0.429	0.002
3	−6079.26	0.930	0.969	0.360	0.513
4	−6045.09	0.936	0.968	0.317	0.002
5	−6010.53	0.937	0.964	0.307	0.507
6	−6097.68	0.949	0.970	0.314	0.146

BIC, Bayesian Information Criterion; AvePP, Average Posterior Probability; ARI, Adjusted Rand Index; BLRT, Bootstrap Likelihood Ratio Test.

Although BIC values improved from the two-profile through the five-profile solution, the more complex solutions did not provide a clearly more parsimonious or interpretable structure in this modest sample and generated smaller additional profiles. This concern was also relevant to the four-profile solution, which included a smaller profile of 19 participants. Specifically, the smallest class included 11 participants in the five-profile solution and 9 participants in the six-profile solution. Alternative 3-, 4-, 5-, and 6-profile solutions are presented in Supplementary Figure 1 and summarized in Supplementary Table 1.

A resampling-based internal validation was conducted using 80% subsampling over 1,000 iterations. The retained two-profile solution showed the highest stability among the examined solutions, with a mean ARI of 0.796 and a median ARI of .866 (IQR = 0.668–0.932). In contrast, the median ARI values were lower for the 3-profile (0.640), 4-profile (0.435), 5-profile (0.370), and 6-profile (0.417) solutions. These stability results are reported in Supplementary Table 2.

**FIGURE 1:**
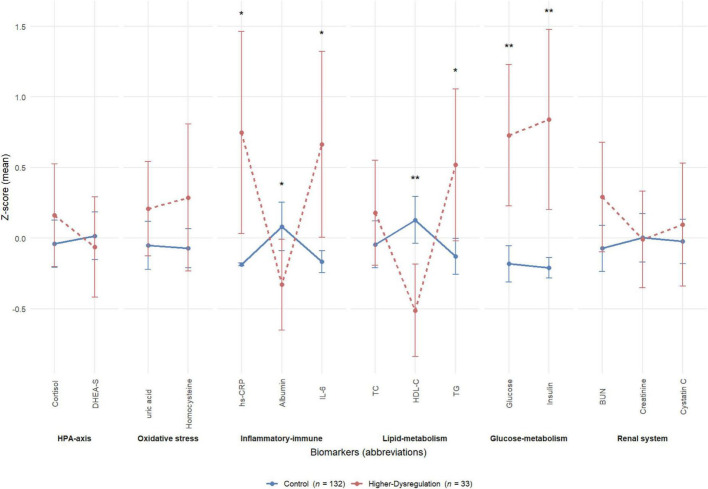
Allostatic load biomarker profiles by latent profile (Control vs. Higher-Dysregulation). DHEA-S, dehydroepiandrosterone sulfate; hs-CRP, high-sensitivity C-reactive protein; IL-6, interleukin-6; TC, total cholesterol; HDL-C, high-density lipoprotein cholesterol; TG, triglycerides; BUN, blood urea nitrogen. Error bars indicate 95% confidence intervals for the group means of the biomarker z-scores. *p < 0.05; **p < 0.01.

Prioritizing parsimony, classification certainty, interpretability, and internal validation, the two-profile solution was retained, comprising a control profile and a higher-dysregulation profile. Profile-specific standardized biomarker patterns are shown in Figure 1. Raw biomarker values by retained AL profile, including means, standard deviations, medians, interquartile ranges, and descriptive mean comparisons, are presented in Supplementary Table 3.

Descriptive statistics and zero-order correlations among the main study variables are presented in Table 2. CD-RISC scores were negatively correlated with BDI scores (*r* = −0.43, *p* < 0.001) and positively correlated with GF:R scores (*r* = 0.19, *p* = 0.017), whereas the correlation with GF:S scores was not statistically significant (*r* = 0.14, *p* = 0.077). BDI scores were negatively correlated with GF:S scores (*r* = −0.36, *p* < 0.001), but not significantly correlated with GF:R scores (*r* = −0.11, *p* = 0.175).

**TABLE 2:** Descriptive statistics and correlations among variables.

Variable	M	SD	1	2	3	4	5	6	7	8
1. Age	23.05	2.59	–							
2. Sex	1.51	0.50	−0.18*	–						
3. SPM	52.38	5.25	−0.13	−0.06	–					
4. AL profile	1.20	0.40	0.01	-0.06	−0.11	–				
5. CD-RISC	65.15	12.98	0.16*	−0.12	−0.07	−0.06	–			
6. BDI	5.19	5.93	0.03	0.10	−0.10	0.21**	−0.43***	–		
7. GF:S	9.14	0.65	−0.09	0.14	0.10	−0.20**	0.14	−0.36***	–	
8. GF:R	9.06	0.57	0.01	0.04	0.06	−0.08	0.19*	−0.11	0.17*	–

SPM, Standard Progressive Matrices; AL, allostatic load; CD-RISC, Connor-Davidson Resilience Scale; BDI, Beck Depression Inventory; GF:S, Global Functioning: Social; GF:R, Global Functioning: Role. Sex was coded as 1 = male and 2 = female. AL profile was coded as 1 = control profile and 2 = higher-dysregulation profile. Pearson correlations are reported.

*p < 0.05;

**p < 0.01;

***p < 0.001.

### 3.2 Moderated regression analyses

Moderated regression analyses examined whether resilience (CD-RISC) moderated associations between AL profile and depressive symptoms (BDI), social functioning (GF:S), and role functioning (GF:R). Descriptive comparisons between profiles are presented in Table 3, and full model estimates are presented in Table 4. Additional comparisons by sex for demographic and main study variables are presented in Supplementary Table 4. Outcome-specific findings are summarized below.

**TABLE 3:** Profile comparisons between control and higher-dysregulation groups.

Variable	Control mean (SD) (*n* = 132)	Higher-dysregulation mean (SD) (*n* = 33)	*t*/χ^2^	*p*
Resilience (CD-RISC)	65.53 (13.14)	63.67 (12.43)	0.74	0.464
Depression (BDI)	4.57 (4.58)	7.70 (9.31)	−1.88 (Welch)	0.069
Social functioning (GF:S)	9.21 (0.58)	8.88 (0.86)	2.61	0.010
Role functioning (GF:R)	9.08 (0.57)	8.97 (0.59)	1.02	0.308
Age	23.05 (2.53)	23.09 (2.87)	−0.09	0.929
SPM	52.67 (5.06)	51.18 (5.87)	1.47	0.145
Sex (% female)	52.3%	45.5%	χ^2^ = 0.26	0.613

BDI, Beck Depression Inventory; CD-RISC, Connor-Davidson Resilience Scale; GF:S, Global Functioning: Social; GF:R, Global Functioning: Role; SPM, Standard Progressive Matrices. Welch’s *t*-test was used when Levene’s test indicated unequal variance.

**TABLE 4:** Moderated regression predicting BDI, GF:S, and GF:R.

Predictor	Outcome	*b* (OLS)	SE	β	*t*	*p*	95% CI
Intercept	BDI	7.793	5.816		1.34	0.182	[−3.987, 19.608]
	GF:S	8.646	0.708		12.21	< 0.001	[7.407, 9.868]
	GF:R	8.636	0.664		13.01	< 0.001	[7.501, 9.952]
Sex (female = 2)	BDI	0.970	0.795	0.082	1.22	0.224	[−0.621, 2.533]
	GF:S	0.159	0.097	0.122	1.64	0.102	[−0.023, 0.345]
	GF:R	0.075	0.091	0.066	0.83	0.409	[−0.096, 0.259]
Age	BDI	0.161	0.156	0.070	1.03	0.304	[−0.106, 0.426]
	GF:S	−0.013	0.019	−0.051	−0.68	0.496	[−0.051, 0.025]
	GF:R	0.000	0.018	−0.002	−0.02	0.981	[−0.029, 0.029]
SPM	BDI	−0.140	0.076	−0.124	−1.85	0.066	[−0.321, 0.033]
	GF:S	0.015	0.009	0.118	1.59	0.114	[−0.005, 0.036]
	GF:R	0.008	0.009	0.072	0.91	0.364	[−0.013, 0.025]
AL profile (Higher- Dysregulation = 2)	BDI	2.227	0.983	0.151	2.27	0.025	[−0.336, 4.898]
	GF:S	−0.234	0.120	−0.144	−1.96	0.052	[−0.481, 0.016]
	GF:R	−0.085	0.112	−0.060	−0.76	0.451	[−0.315, 0.139]
Resilience (CD-RISC) (mean-centered)	BDI	−0.138	0.034	−0.303	−4.09	< 0.001	[−0.199, −0.079]
	GF:S	0.001	0.004	0.025	0.31	0.759	[−0.006, 0.008]
	GF:R	0.009	0.004	0.210	2.39	0.018	[0.003, 0.016]
AL profile × resilience	BDI	−0.335	0.079	−0.312	−4.26	<0.001	[−0.645, −0.055]
	GF:S	0.038	0.010	0.318	3.92	< 0.001	[0.003, 0.063]
	GF:R	−0.004	0.009	−0.034	−0.39	0.696	[−0.024, 0.015]

BDI, Beck Depression Inventory; GF:S, Global Functioning: Social; GF:R, Global Functioning: Role; SPM, Standard Progressive Matrices; CD-RISC, Connor-Davidson Resilience Scale. Primary estimates are unstandardized ordinary least squares (OLS) coefficients (*b*), and standardized coefficients (β) are provided for comparability. Statistical significance was determined using the 95% bootstrap confidence interval (*R* = 5,000); model-based *p*-values are reported for reference. All models included age, sex (female = 2), and SPM as covariates. AL profile dummy (1 = Control, 2 = Higher-Dysregulation); the table label “AL profile (Higher-Dysregulation = 2)” reflects this coding.

#### 3.2.1 Depressive symptoms (BDI)

**FIGURE 2:**
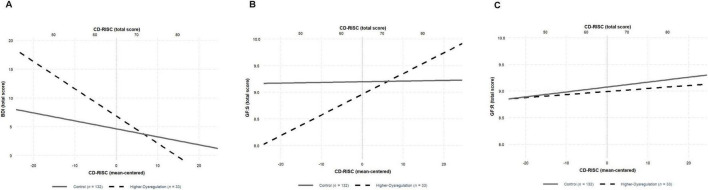
Interaction between AL profile and resilience (CD-RISC) predicting **(A)** depressive symptoms (BDI), **(B)** social functioning (GF:S), and **(C)** role functioning (GF:R). BDI, Beck Depression Inventory; CD-RISC, Connor-Davidson Resilience Scale; GF:S, Global Functioning: Social; GF:R, Global Functioning: Role. Regression lines display predicted means with covariates [age, sex, and Standard Progressive Matrices (SPM)] held at their sample means. CD-RISC is mean-centered; therefore, 0 on the x-axis corresponds to the sample mean.

The regression model predicting depressive symptoms was significant, *F*(6, 158) = 12.66, *p* < 0.001, *R*^2^ = 0.325 (adjusted *R*^2^ = 0.299). Although the AL profile effect was statistically significant in the model (*b* = 2.227, *p* = 0.025), it did not meet the pre-specified criterion based on percentile bootstrap confidence intervals [95% CI (−0.336, 4.898)]. This divergence can occur because conventional OLS *p*-values rely on parametric standard-error assumptions, whereas percentile bootstrap confidence intervals are derived from the empirical resampling distribution; accordingly, the bootstrap confidence interval was retained as the primary inferential criterion. Resilience (CD-RISC) was negatively associated with depressive symptoms [*b* = −0.138, *p* < 0.001, 95% CI (−0.199, −0.079)]. The AL profile × resilience interaction was significant [*b* = −0.335, *p* < 0.001, 95% CI (−0.645, −0.055); Figure 2A]. Simple slopes analyses indicated that higher resilience was associated with lower depressive symptoms in both profiles, with a stronger effect in the higher-dysregulation profile [control: *b* = −0.138, *p* < 0.001, 95% CI (−0.199, −0.079); higher-dysregulation: *b* = −0.474, *p* < 0.001, 95% CI (−0.777, −0.199)]. One observation exceeded the Cook’s distance threshold (0.5); its exclusion did not alter the results.

#### 3.2.2 Social functioning (GF:S)

The model predicting social functioning was statistically significant, *F*(6, 158) = 5.48, *p* < 0.001, *R*^2^ = .172 (adjusted *R*^2^ = 0.141). A significant AL profile × resilience interaction was identified [*b* = 0.038, *p* < 0.001, 95% CI (0.003, 0.063); Figure 2B]. Simple slope analysis showed that resilience was not associated with social functioning in the control group [*b* = 0.001, *p* = 0.759, 95% CI (-0.006, 0.008)], but was positively associated in the higher-dysregulation group [*b* = 0.039, *p* < 0.001, 95% CI (0.005, 0.062)].

#### 3.2.3 Role functioning (GF:R)

The model predicting role functioning was not statistically significant, *F*(6, 158) = 1.36, *p* = 0.234, *R*^2^ = 0.049 (adjusted *R*^2^ = 0.013). Resilience showed a small but significant main effect [*b* = 0.009, *p* = 0.018, 95% CI (0.003, 0.016)], whereas the AL profile × resilience interaction was not significant [*b* = −0.004, *p* = 0.696, 95% CI (−0.024, 0.015); Figure 2C].

## 4 Discussion

In the current study of young adults, LPA of 15 physiological biomarkers identified a two-profile solution (control vs. higher-dysregulation) based on model fit (BIC, BLRT), classification quality (entropy, AvePP), and stability (ARI). The higher-dysregulation profile showed broadly elevated physiological dysregulation, with pronounced deviations in inflammatory-immune and lipid/glucose-metabolism systems (Figure 1). These profiles are best interpreted as relative patterns of biomarker dysregulation within this nonclinical sample rather than as clinically validated risk categories. Accordingly, the higher-dysregulation profile should not be equated with clinically abnormal biomarker values or validated risk for hard clinical endpoints such as hospitalization or mortality.

The person-centered approach provided a descriptive framework for summarizing co-occurring biomarker patterns, consistent with previous latent class/profile-based AL research describing heterogeneity in multisystem physiological dysregulation (Forrester et al., 2019). The prominence of inflammatory-immune and lipid/glucose-metabolic deviations is also consistent with evidence linking inflammatory and metabolic dysregulation to depression-related outcomes (Milaneschi et al., 2020; Carbone, 2021). At the same time, conventional AL indices remain widely used and provide an important cumulative summary of multisystem biological risk, although their construction involves methodological choices such as biomarker selection, thresholds, and weighting (Seeman et al., 2001; Juster et al., 2010; Beese et al., 2022). Because the present study did not directly compare LPA with conventional AL index-based scoring, the findings do not provide evidence that LPA is superior to index-based AL operationalization’s. Rather, the present results suggest that LPA may be useful for describing heterogeneity in multisystem biomarker patterns, while future studies should directly compare profile-based and index-based approaches.

For depressive symptoms, the primary finding was a significant AL profile × resilience interaction. Higher resilience was associated with lower depressive symptoms in both profiles, with a stronger association in the higher-dysregulation group. This pattern is consistent with a stress-buffering model, in which protective resources show stronger associations with outcomes under higher levels of stress or adversity (Cohen and Wills, 1985). Supporting this interpretation, studies of individuals from low socioeconomic backgrounds have shown that adaptive coping strategies (e.g., “shift-and-persist”) are associated with lower AL, particularly under high adversity (Chen et al., 2012). Similarly, research on daily stress and bereavement demonstrates that resilience-related factors, such as positive affect, moderate stress reactivity and facilitate recovery (Ong et al., 2006). Together, these findings indicate that the association between resilience and lower depressive symptoms was stronger among individuals with relatively greater physiological dysregulation.

A similar, though more modest, pattern was observed for social functioning. The AL profile × resilience interaction indicated that resilience was more strongly associated with better social functioning in the higher-dysregulation group. This is consistent with prior work linking higher resilience to improved psychosocial functioning (Dou et al., 2022; Echezarraga et al., 2022), and higher AL to poorer functional and social outcomes (Karlamangla et al., 2002; Gallagher et al., 2021). Given that the GF:S scale directly assesses interpersonal relationships and social engagement, resilience-related capacities, such as effective coping, emotion regulation, and prosocial behavior, may be reflected in better social functioning (Cornblatt et al., 2007). Nevertheless, resilience and social functioning are conceptually related constructs, and the present findings are more cautiously described as associations between related but distinct measures rather than as evidence of causal improvement in functioning. These findings should also be interpreted in light of construct overlap among the measures. Some CD-RISC items assess perceived coping capacity, emotional recovery, and persistence under stress, which may overlap conceptually and in item content with BDI items reflecting low affect and helplessness, as well as with GF:S ratings of interpersonal engagement and relationship quality. Therefore, the significant moderation effects should not be interpreted as evidence that resilience is fully independent of depressive symptoms or social functioning, or as demonstrating causal improvement in functioning. Rather, they indicate that the observed associations between measured psychological resilience and outcomes differed by AL profile in this sample. Future studies should reduce this ambiguity by assessing resilience and outcomes at independent measurement occasions, incorporating multi-informant or behavioral measures, and applying latent variable models that separate shared variance from construct-specific variance.

In contrast, the moderation model for role functioning was not significant. This may reflect both conceptual and distributional factors. Conceptually, GF:R assesses performance in structured domains (e.g., school, work), which are more strongly influenced by environmental and contextual constraints than interpersonal functioning (Carrión et al., 2019). Distributionally, both GF measures showed upper-end clustering with limited variability, and no between-profile difference was observed for GF:R. These factors may have reduced statistical power to detect interaction effects due to range restriction and potential ceiling effects (McClelland and Judd, 1993).

Supplementary comparisons by sex indicated that there were no significant differences in resilience, depressive symptoms, social functioning, or role functioning. This null pattern may be understood in the context of prior evidence linking sex and gender to AL through biological, behavioral, and social pathways (Seeman et al., 2002; Volarić et al., 2024); in the present sample, the absence of significant sex differences may reflect the young, nonclinical, and relatively homogeneous sample, modest sample size, and limited variability in psychological and functional outcomes. Beyond these descriptive findings, future AL research may benefit from considering sex and gender as contextual factors. Future prevention-oriented studies with larger samples could examine whether modifiable factors, such as sleep, physical activity, metabolic health-related behaviors, stress management, and social support, are differentially associated with AL profiles, resilience, and adaptation across sex and gender contexts. Epigenetic aging was not assessed in the present study; therefore, the relationship of epigenetic aging with AL profiles and resilience should be examined directly in future longitudinal research incorporating biological aging indicators, including DNA methylation-based measures.

Several limitations should be considered when interpreting these findings. First, the biomarker panel did not include cardiovascular and autonomic indicators (e.g., blood pressure, heart rate variability) or diurnal measures (e.g., cortisol slope), which may provide additional insight into AL. Second, although the two-profile solution was selected based on parsimony and classification quality, alternative model specifications may yield different structures, and some misclassification is possible. The sample size was modest relative to the number of biomarkers included in the LPA, and the higher-dysregulation profile included only 33 participants. Therefore, the retained profile structure and the profile-specific moderation findings warrant cautious interpretation and replication in larger samples. Third, the sample comprised nonclinical young adults from a single country and was modest in size, limiting generalizability and sensitivity to smaller effects. Fourth, the GF:S and GF:R scales showed near-ceiling distributions in this nonclinical sample, with limited variability in both functioning outcomes. This represents a measurement-related design-level constraint that may have reduced power to detect associations and interactions for both social and role functioning, including the significant but modest social functioning finding. In addition, the GF:S and GF:R scales were originally developed and validated for clinical and subclinical populations, which may limit their sensitivity in a healthy convenience sample. Fifth, although analyses adjusted for age, sex, and cognitive ability, several potentially important confounders were not comprehensively assessed or included in the models, including socioeconomic status, body mass index, sleep, physical activity, substance use, medication use, recent illness, and acute inflammatory states. These factors may influence both biomarker levels and psychosocial outcomes. Sixth, pre-collection conditions for biomarker assessment were not fully standardized or comprehensively assessed. Blood samples were collected during broad morning or afternoon collection windows, and an additional sensitivity check indicated that AL profile distribution was not detectably imbalanced by broad blood draw period, χ^2^(1, 165) = 0.06, *p* = 0.806, φ = 0.02. However, exact blood draw timing within each collection window and fasting status were not uniformly standardized. In addition, recent infection or acute inflammatory symptoms, medication use at the time of blood collection, acute exercise, and sleep before blood collection were not systematically available in a form that allowed biomarker-specific adjustment or exclusion. Therefore, residual confounding related to pre-collection conditions cannot be ruled out. Cortisol, inflammatory markers, and metabolic biomarkers are sensitive to circadian, behavioral, and acute inflammatory influences; therefore, these factors may have affected absolute biomarker values and potentially profile classification. Future research should incorporate broader biomarker panels, preregistered sensitivity analyses for LPA, longitudinal or experimental designs, and more diverse samples to strengthen causal inference and generalizability.

In summary, resilience showed a stronger inverse association with depressive symptoms under conditions of greater physiological dysregulation. A similar, though more modest, pattern was observed for social functioning, whereas no interaction was found for role functioning. These findings suggest that psychological resources may be more closely associated with social and emotional adaptation among individuals with relatively greater physiological dysregulation, whereas role functioning may depend more on structural and contextual factors. Accordingly, resilience-related processes may be relevant for supporting adaptation in young adults with elevated physiological burden, although implications for intervention should be interpreted cautiously given the cross-sectional design.

## Data Availability

The raw data supporting the conclusions of this article will be made available by the authors, without undue reservation.

## References

[B1] Arnett J. J. Žukauskienë R. Sugimura K. (2014). The new life stage of emerging adulthood at ages 18–29 years: Implications for mental health. *Lancet Psychiatry* 1 569–576. 10.1016/s2215-0366(14)00080-7 26361316

[B2] Atkinson L. Joshi D. Raina P. Griffith L. E. MacMillan H. Gonzalez A. (2023). Social engagement and allostatic load mediate between adverse childhood experiences and multimorbidity in mid to late adulthood: The Canadian longitudinal study on aging. *Psychol. Med.* 53 1437–1447. 10.1017/s0033291721003019 37010223 PMC10009404

[B3] Baek H.-S. Lee K.-U. Joo E.-J. Lee M.-Y. Choi K.-S. (2010). Reliability and validity of the Korean version of the connor–davidson resilience scale. *Psychiatry Invest.* 7 109–115. 10.4306/pi.2010.7.2.109 20577619 PMC2890864

[B4] Beck A. T. Steer R. A. Garbin M. G. (1988). Psychometric properties of the beck depression inventory: Twenty-five years of evaluation. *Clin. Psychol. Rev.* 8 77–100. 10.1016/0272-7358(88)90050-5

[B5] Beese S. Postma J. Graves J. M. (2022). Allostatic load measurement: A systematic review of reviews, database inventory, and considerations for neighborhood research. *Int. J. Environ. Res. Public Health* 19:17006. 10.3390/ijerph192417006 36554888 PMC9779615

[B6] Belsley D. A. Kuh E. Welsch R. E. (1980). *Regression Diagnostics: Identifying Influential Data and Sources of Collinearity.* New York: Wiley-Interscience.

[B7] Brody G. H. Yu T. Chen E. Miller G. E. Kogan S. M. Beach S. R. H. (2013). Is resilience only skin deep? Rural African Americans’ socioeconomic status–related risk and competence in preadolescence and psychological adjustment and allostatic load at age 19. *Psychol. Sci.* 24 1285–1293. 10.1177/0956797612471954 23722980 PMC3713113

[B8] Brooks K. P. Gruenewald T. Karlamangla A. Hu P. Koretz B. Seeman T. E. (2014). Social relationships and allostatic load in the MIDUS study. *Health Psychol.* 33 1373–1381. 10.1037/a0034528 24447186 PMC4104264

[B9] Carbone J. T. (2021). Allostatic load and mental health: A latent class analysis of physiological dysregulation. *Stress* 24 394–403. 10.1080/10253890.2020.1813711 32835575

[B10] Carrión R. E. Auther A. M. McLaughlin D. Olsen R. Addington J. Bearden C. E. et al. (2019). The global functioning: social and role scales—further validation in a large sample of adolescents and young adults at clinical high risk for psychosis. *Schizophrenia Bull.* 45 763–772. 10.1093/schbul/sby126 30351423 PMC6581127

[B11] Celeux G. Soromenho G. (1996). An entropy criterion for assessing the number of clusters in a mixture model. *J. Classification* 13 195–212. 10.1007/bf01246098

[B12] Chen E. Miller G. E. Lachman M. E. Gruenewald T. L. Seeman T. E. (2012). Protective factors for adults from low-childhood socioeconomic circumstances: The benefits of shift-and-persist for allostatic load. *Psychosomatic Med.* 74 178–186. 10.1097/PSY.0b013e31824206fd 22286848 PMC3273596

[B13] Cohen S. Wills T. A. (1985). Stress, social support, and the buffering hypothesis. *Psychol. Bull.* 98 310–357. 10.1037/0033-2909.98.2.310 3901065

[B14] Connor K. M. Davidson J. R. T. (2003). Development of a new resilience scale: The Connor–davidson resilience scale (CD-RISC). *Depression Anxiety* 18 76–82. 10.1002/da.10113 12964174

[B15] Cook R. D. (1977). Detection of influential observation in linear regression. *Technometrics* 19 15–18. 10.1080/00401706.1977.10489493

[B16] Cornblatt B. A. Auther A. M. Niendam T. Smith C. W. Zinberg J. Bearden C. E. et al. (2007). Preliminary findings for two new measures of social and role functioning in the prodromal phase of schizophrenia. *Schizophrenia Bull.* 33 688–702. 10.1093/schbul/sbm029 17440198 PMC2526147

[B17] de Oliveira C. Sabbah W. Bernabé E. (2023). Allostatic load and depressive symptoms in older adults: An analysis of 12-year panel data. *Psychoneuroendocrinology* 152:106100. 10.1016/j.psyneuen.2023.106100 36989564

[B18] Dou W. Yu X. Fang H. Lu D. Cai L. Zhu C. et al. (2022). Family and psychosocial functioning in bipolar disorder: The mediating effects of social support, resilience and suicidal ideation. *Front. Psychol.* 12:807546. 10.3389/fpsyg.2021.807546 35153929 PMC8832135

[B19] Echezarraga A. Calvete E. Orue I. Las Hayas C. (2022). Resilience moderates the associations between bipolar disorder mood episodes and mental health. *Clínica y Salud*. 33 83–90. 10.5093/clysa2022a8

[B20] Efron B. Tibshirani R. J. (1993). *An Introduction to the Bootstrap.* New York: Chapman & Hall/CRC.

[B21] Färber F. Rosendahl J. (2018). The association between resilience and mental health in the somatically Ill: A systematic review and meta-analysis. *Deutsches Ärzteblatt Int.* 115 621–627. 10.3238/arztebl.2018.0621 30373706 PMC6218704

[B22] Forrester S. N. Leoutsakos J. M. Gallo J. J. Thorpe R. J. Seeman T. E. (2019). Association between allostatic load and health behaviours: A latent class approach. *J. Epidemiol. Community Health* 73 340–345. 10.1136/jech-2018-211289 30700494

[B23] Gallagher S. Muldoon O. T. Bennett K. M. (2021). Multiple group membership, social network size, allostatic load and well-being: A mediation analysis. *J. Psychosomatic Res.* 151:110636. 10.1016/j.jpsychores.2021.110636 34638016 PMC8660054

[B24] Gou Y. Cheng S. Kang M. Zhou R. Liu C. Hui J. et al. (2025). Association of allostatic load with depression, anxiety, and suicide: A prospective cohort study. *Biol. Psychiatry* 97 786–793. 10.1016/j.biopsych.2024.09.026 39395472

[B25] Guidi J. Lucente M. Sonino N. Fava G. A. (2020). Allostatic load and its impact on health: A systematic review. *Psychotherapy Psychosomat.* 90 11–27. 10.1159/000510696 32799204

[B26] Honkalampi K. Virtanen M. Hintsa T. Ruusunen A. Mäntyselkä P. Ali-Sisto T. et al. (2021). Comparison of the level of allostatic load between patients with major depression and the general population. *J. Psychosomatic Res.* 143:110389. 10.1016/j.jpsychores.2021.110389 33609985

[B27] Hu T. Zhang D. Wang J.-L. (2015). A meta-analysis of the trait resilience and mental health. *Pers. Individ. Dif.* 76 18–27. 10.1016/j.paid.2014.11.039

[B28] Hubert L. Arabie P. (1985). Comparing partitions. *J. Classification* 2 193–218. 10.1007/bf01908075

[B29] Johnson A. J. Dudley W. N. Wideman L. Schulz M. (2019). Physiological risk profiles and allostatic load: Using latent profile analysis to examine socioeconomic differences in physiological patterns of risk. *Eur. J. Environ. Public Health* 3:em0029. 10.29333/ejeph/5870 42450140

[B30] Juster R.-P. McEwen B. S. Lupien S. J. (2010). Allostatic load biomarkers of chronic stress and impact on health and cognition. *Neurosci. Biobehav. Rev.* 35 2–16. 10.1016/j.neubiorev.2009.10.002 19822172

[B31] Karlamangla A. S. Singer B. H. McEwen B. S. Rowe J. W. Seeman T. E. (2002). Allostatic load as a predictor of functional decline: MacArthur studies of successful aging. *J. Clin. Epidemiol.* 55 696–710. 10.1016/s0895-4356(02)00399-2 12160918

[B32] Kobrosly R. W. van Wijngaarden E. Seplaki C. L. Cory-Slechta D. A. Moynihan J. (2014). Depressive symptoms are associated with allostatic load among community-dwelling older adults. *Physiol. Behav.* 123 223–230. 10.1016/j.physbeh.2013.10.014 24432360 PMC3934832

[B33] Lenart-Bugla M. Szcześniak D. Bugla B. Kowalski K. Niwa S. Rymaszewska J. et al. (2022). The association between allostatic load and brain: A systematic review. *Psychoneuroendocrinology* 145:105917. 10.1016/j.psyneuen.2022.105917 36113380

[B34] Liang Y. Booker C. (2024). Allostatic load and chronic pain: A prospective finding from the national survey of midlife development in the United States, 2004–2014. *BMC Public Health* 24:416. 10.1186/s12889-024-17888-1 38336697 PMC10854121

[B35] Liston C. McEwen B. S. Casey B. J. (2009). Psychosocial stress reversibly disrupts prefrontal processing and attentional control. *Proc. Natl. Acad. Sci. U S A.* 106 912–917. 10.1073/pnas.0807041106 19139412 PMC2621252

[B36] Long J. A. (2024). *Interactions: Comprehensive, User-Friendly Toolkit for Probing Interactions.* Vienna: Comprehensive R Archive Network (CRAN).

[B37] Lucarini V. Kazes M. Krebs E. Morin V. Godignon M. De Gasquet M. et al. (2024). Validation of the French version of the global functioning: Social and global functioning: Role scales in adolescents and young adults seeking help in early intervention clinics. *Early Intervent. Psychiatry* 18 3–9. 10.1111/eip.13427 37037927

[B38] Masyn K. E. (2013). “Latent class analysis and finite mixture modeling,” in *The Oxford Handbook of Quantitative Methods*, ed. Little T. D. (New York: Oxford University Press), 551–611. 10.1093/oxfordhb/9780199934898.013.0025

[B39] McClelland G. H. Judd C. M. (1993). Statistical difficulties of detecting interactions and moderator effects. *Psychol. Bull.* 114 376–390. 10.1037/0033-2909.114.2.376 8416037

[B40] McCrory C. McLoughlin S. Layte R. Ní Cheallaigh C. O’Halloran A. M. Barros H. et al. (2023). Towards a consensus definition of allostatic load: A multi-cohort, multi-system, multi-biomarker individual participant data (IPD) meta-analysis. *Psychoneuroendocrinology* 153:106117. 10.1016/j.psyneuen.2023.106117 37100008 PMC10620736

[B41] McEwen B. S. (2012). Brain on stress: How the social environment gets under the skin. *Proc. Natl. Acad. Sci. U S A.* 109 17180–17185. 10.1073/pnas.1121254109 23045648 PMC3477378

[B42] McEwen B. S. Stellar E. (1993). Stress and the individual: Mechanisms leading to disease. *Arch. Internal Med.* 153 2093–2101. 10.1001/archinte.1993.00410180039004 8379800

[B43] McLachlan G. J. Peel D. (2000). *Finite Mixture Models.* New York: Wiley-Interscience.

[B44] McLoughlin S. Kenny R. A. McCrory C. (2020). Does the choice of allostatic load scoring algorithm matter for predicting age-related health outcomes? *Psychoneuroendocrinology* 120:104789. 10.1016/j.psyneuen.2020.104789 32739647

[B45] Milaneschi Y. Lamers F. Berk M. Penninx B. W. J. H. (2020). Depression heterogeneity and its biological underpinnings: Toward immunometabolic depression. *Biol. Psychiatry* 88 369–380. 10.1016/j.biopsych.2020.01.014 32247527

[B46] Miller G. E. Brody G. H. Yu T. Chen E. (2014). A family-oriented psychosocial intervention reduces inflammation in low-SES African American youth. *Proc. Natl. Acad. Sci. U S A.* 111 11287–11292. 10.1073/pnas.1406578111 25049403 PMC4128159

[B47] Miller G. E. Yu T. Chen E. Brody G. H. (2015). Self-control forecasts better psychosocial outcomes but faster epigenetic aging in low-SES youth. *Proc. Natl. Acad. Sci. U S A.* 112 10325–10330. 10.1073/pnas.1505063112 26170291 PMC4547243

[B48] Nylund K. L. Asparouhov T. Muthén B. O. (2007). Deciding on the number of classes in latent class analysis and growth mixture modeling: A Monte Carlo simulation study. *Struct. Eq. Model. Multidisciplinary J.* 14 535–569. 10.1080/10705510701575396

[B49] Nylund-Gibson K. Choi A. Y. (2018). Ten frequently asked questions about latent class analysis. *Transl. Issues Psychol. Sci.* 4 440–461. 10.1037/tps0000176

[B50] O’Brien R. M. (2007). A caution regarding rules of thumb for variance inflation factors. *Quality Quantity* 41 673–690. 10.1007/s11135-006-9018-6

[B51] Ong A. D. Bergeman C. S. Bisconti T. L. Wallace K. A. (2006). Psychological resilience, positive emotions, and successful adaptation to stress in later life. *J. Pers. Soc. Psychol.* 91 730–749. 10.1037/0022-3514.91.4.730 17014296

[B52] Pfaltz M. C. Schnyder U. (2023). Allostatic load and allostatic overload: Preventive and clinical implications. *Psychotherapy Psychosomat.* 92 279–282. 10.1159/000534340 37931612 PMC10716872

[B53] Raven J. (2000). The Raven’s progressive matrices: Change and stability over culture and time. *Cogn. Psychol.* 41 1–48. 10.1006/cogp.1999.0735 10945921

[B54] Raven J. C. Court J. H. Raven J. (1990). *Manual for Raven’s Progressive Matrices and Vocabulary Scales: Standard Progressive Matrices.* Oxford: Oxford Psychologists Press.

[B55] Rhee M. K. Lee Y. H. Park S. H. Sohn C. H. Hong S. K. Lee B. K. et al. (1995). A standardization study of beck depression inventory 1–Korean version (K-BDI): Reliability and factor analysis. *Korean J. Psychopathol.* 4 77–95.

[B56] Schwarz G. (1978). Estimating the dimension of a model. *Ann. Stat.* 6 461–464. 10.1214/aos/1176344136

[B57] Scrucca L. Fop M. Murphy T. B. Raftery A. E. (2016). mclust 5: Clustering, classification and density estimation using Gaussian finite mixture models. *R J.* 8 289–317. 10.32614/RJ-2016-021 27818791 PMC5096736

[B58] Seeman T. E. Singer B. H. Rowe J. W. Horwitz R. I. McEwen B. S. (2001). Allostatic load as a marker of cumulative biological risk: MacArthur studies of successful aging. *Proc. Natl. Acad. Sci. U S A.* 98 4770–4775. 10.1073/pnas.081072698 11287659 PMC31909

[B59] Seeman T. E. Singer B. H. Ryff C. D. Dienberg Love G. Levy-Storms L. (2002). Social relationships, gender, and allostatic load across two age cohorts. *Psychosomatic Med.* 64 395–406. 10.1097/00006842-200205000-00004 12021414

[B60] Seeman T. Glei D. Goldman N. Weinstein M. Singer B. Lin Y.-H. (2004). Social relationships and allostatic load in Taiwanese elderly and near elderly. *Soc. Sci. Med.* 59 2245–2257. 10.1016/j.socscimed.2004.03.027 15450701

[B61] Sheehan D. V. Lecrubier Y. Sheehan K. H. Amorim P. Janavs J. Weiller E. et al. (1998). The Mini-international neuropsychiatric interview (MINI): The development and validation of a structured diagnostic psychiatric interview for DSM-IV and ICD-10. *J. Clin Psychiatry* 59 22–33. 9881538

[B62] Sterling P. Eyer J. (1988). “Allostasis: A new paradigm to explain arousal pathology,” in *Handbook of Life Stress, Cognition and Health*, eds Fisher S. J. Reason J. (New York: Wiley), 629–649.

[B63] Tein J.-Y. Coxe S. Cham H. (2013). Statistical power to detect the correct number of classes in latent profile analysis. *Struct. Eq. Model. Multidisciplinary J.* 20 640–657. 10.1080/10705511.2013.824781 24489457 PMC3904803

[B64] Volarić N. Šojat D. Volarić M. Vèev I. Keškić T. Majnarić L. T. (2024). The gender and age perspectives of allostatic load. *Front. Med.* 11:1502940. 10.3389/fmed.2024.1502940 39741506 PMC11685202

[B65] Wambua G. N. Kilian S. Ntlantsana V. Chiliza B. (2020). The association between resilience and psychosocial functioning in schizophrenia: A systematic review and meta-analysis. *Psychiatry Res.* 293:113374. 10.1016/j.psychres.2020.113374 32795771

[B66] Wermelinger, Ávila M. P. Granero Lucchetti A. L. Lucchetti G. (2017). Association between depression and resilience in older adults: A systematic review and meta-analysis. *Int. J. Geriatr. Psychiatry* 32 237–246. 10.1002/gps.4619 27805730

[B67] Wiley J. F. Bei B. Bower J. E. Stanton A. L. (2017). Relationship of psychosocial resources with allostatic load: A systematic review. *Psychosomatic Med.* 79 283–292. 10.1097/PSY.0000000000000395 27768647 PMC5374015

[B68] Yoo S.-W. Kim Y.-S. Noh J.-S. Oh K.-S. Kim C.-H. NamKoong K. et al. (2006). Validity of Korean version of the mini-international neuropsychiatric interview. *Anxiety Mood* 2 50–55.

[B69] Zhao W. Si Y. Li X. Zhao Y. Jia S. Dong B. (2024). Association of allostatic load with functional disability in the China health and retirement longitudinal study. *J. Nutr. Health Aging* 28:100367. 10.1016/j.jnha.2024.100367 39341031 PMC12879250

[B70] Zhao X. Tong S. Yang Y. (2022). The correlation between quality of life and positive psychological resources in cancer patients: A meta-analysis. *Front. Psychol.* 13:883157. 10.3389/fpsyg.2022.883157 35783766 PMC9245894

[B71] Zilioli S. Slatcher R. B. Ong A. D. Gruenewald T. L. (2015). Purpose in life predicts allostatic load ten years later. *J. Psychosomatic Res.* 79 451–457. 10.1016/j.jpsychores.2015.09.013 26526322 PMC4684637

[B72] Zuur A. F. Ieno E. N. Elphick C. S. (2010). A protocol for data exploration to avoid common statistical problems. *Methods Ecol. Evol.* 1 3–14. 10.1111/j.2041-210X.2009.00001.x

