# Supplementary material for: Allostatic load profiles, resilience, and psychosocial adaptation in young adults
Source: Front Psychol. 2026 Jul 30;17:1860265. doi: 10.3389/fpsyg.2026.1860265 (PMC13469248; doi:10.3389/fpsyg.2026.1860265)
Supplement: Supplementary file 1 [file Table_1.docx]

**Supplementary Materials**

*Allostatic Load Profiles, Resilience, and Psychosocial Adaptation in Young Adults*

This supplementary document provides additional information supporting the latent profile analysis of allostatic load biomarkers. Supplementary Table S1 summarizes the alternative 3-, 4-, 5-, and 6-profile solutions, Supplementary Table S2 presents subsampling-based stability results for the 2- to 6-profile solutions, Supplementary Table S3 reports raw biomarker values and descriptive mean comparisons by the retained two-profile solution, and Supplementary Table S4 presents supplementary comparisons by sex for demographic and main study variables. The retained two-profile solution showed the highest mean and median adjusted Rand index, whereas more complex solutions produced progressively smaller subgroups. Supplementary Figure S1 illustrates standardized biomarker patterns for the alternative profile solutions. These materials are provided to support interpretation of model enumeration, profile characterization, and supplementary comparisons by sex and should be interpreted as supplementary descriptive evidence rather than independent confirmatory analyses.

**Supplementary Table S1.** Alternative latent profile solution characteristics (*n* = 165)

| Solution | Class | *n* | % | Descriptive profile label |
| --- | --- | --- | --- | --- |
| 3-profile | 1 | 68 | 41.21 | Renal/oxidative elevation profile |
| 3-profile | 2 | 72 | 43.64 | Lower-dysregulation/high-HDL profile |
| 3-profile | 3 | 25 | 15.15 | Inflammatory-metabolic dysregulation profile |
| 4-profile | 1 | 61 | 36.97 | Renal/oxidative elevation profile |
| 4-profile | 2 | 44 | 26.67 | Lower-dysregulation/high-HDL profile |
| 4-profile | 3 | 41 | 24.85 | Mild glucose-insulin elevation profile |
| 4-profile | 4 | 19 | 11.52 | Pronounced inflammatory-metabolic dysregulation profile |
| 5-profile | 1 | 20 | 12.12 | Inflammatory-marker elevation profile |
| 5-profile | 2 | 55 | 33.33 | Renal/oxidative elevation profile |
| 5-profile | 3 | 38 | 23.03 | Lower-dysregulation/high-HDL profile |
| 5-profile | 4 | 41 | 24.85 | Mild glucose-insulin elevation profile |
| 5-profile | 5 | 11 | 6.67 | Pronounced inflammatory-metabolic dysregulation profile |
| 6-profile | 1 | 24 | 14.55 | Mild inflammatory elevation/high-HDL profile |
| 6-profile | 2 | 36 | 21.82 | Renal/HPA-axis elevation profile |
| 6-profile | 3 | 34 | 20.61 | Renal/oxidative-lipid dysregulation profile |
| 6-profile | 4 | 44 | 26.67 | Lower-dysregulation/high-HDL profile |
| 6-profile | 5 | 18 | 10.91 | Mild glucose-insulin elevation profile |
| 6-profile | 6 | 9 | 5.45 | Pronounced inflammatory-metabolic dysregulation profile |

**Note.** Labels for the alternative profiles are descriptive and were assigned to summarize the dominant biomarker pattern within each non-retained solution. These labels were used to aid interpretation of the alternative solutions and were not intended to indicate clinically validated categories.

**Supplementary Table S2.** Subsampling-based stability of the 2- to 6-profile solutions

| Profiles | ARI mean  (SD) | ARI median  [IQR] | Median entropy | Median AvePP | Median smallest profile proportion |
| --- | --- | --- | --- | --- | --- |
| 2 | .796 (.177) | .866 [.668-.932] | .968 | .993 | .197 |
| 3 | .602 (.213) | .640 [.435-.779] | .937 | .974 | .114 |
| 4 | .448 (.152) | .435 [.330-.556] | .945 | .973 | .076 |
| 5 | .391 (.120) | .370 [.301-.454] | .951 | .973 | .068 |
| 6 | .423 (.096) | .417 [.359-.482] | .957 | .975 | .061 |

**Note.** Stability was evaluated using 80% subsampling repeated 1,000 times. In each iteration, the same number of profiles was re-estimated in the subsample, and ARI, entropy, AvePP, and the smallest profile proportion were calculated. Values are summarized across iterations as mean (SD) or median [IQR]. Abbreviations: ARI, adjusted Rand index; AvePP, average posterior probability.

**Supplementary Table S3.** Raw biomarker values and mean comparisons by retained allostatic load profile

| Biomarker | Unit | Control mean (SD)  (*n* = 132) | Control median [IQR]  (*n* = 132) | Higher-dysregulation mean (SD) (*n* = 33) | Higher-dysregulation median [IQR] (*n* = 33) | *t* | *p* |
| --- | --- | --- | --- | --- | --- | --- | --- |
| Cortisol | µg/dL | 8.69 (3.43) | 8.45 [6.00-10.68] | 9.39 (3.71) | 8.60 [7.00-12.10] | 1.04 | .301 |
| DHEA-S | µg/dL | 303.49 (110.17) | 290.00 [232.00-371.50] | 294.76 (115.46) | 293.00 [229.00-337.00] | -0.40 | .687 |
| Uric acid | mg/dL | 5.31 (1.48) | 5.10 [4.28-6.30] | 5.70 (1.45) | 5.50 [4.50-6.60] | 1.34 | .181 |
| Homocysteine | µmol/L | 16.34 (4.59) | 15.75 [12.55-19.26] | 18.37 (8.65) | 16.49 [14.71-21.12] | 1.86 | .065 |
| hs-CRP | mg/L | 0.44 (0.30) | 0.35 [0.24-0.53] | 5.12 (10.51) | 2.30 [0.72-3.78] | 2.56  (Welch) | .015 |
| Albumin | g/dL | 4.76 (0.21) | 4.80 [4.60-4.90] | 4.67 (0.19) | 4.60 [4.50-4.80] | -2.14 | .034 |
| IL-6 | pg/mL | 1.69 (1.69) | 0.86 [0.66-1.77] | 4.78 (7.19) | 2.15 [1.03-5.12] | 2.46  (Welch) | .019 |
| TC | mg/dL | 174.09 (32.66) | 171.00 [155.75-187.00] | 181.61 (36.52) | 183.00 [153.00-212.00] | 1.15 | .250 |
| HDL-C | mg/dL | 58.89 (12.75) | 57.00 [50.00-68.00] | 50.52 (12.54) | 48.00 [40.00-61.00] | -3.39 | <.001 |
| TG | mg/dL | 98.25 (50.39) | 86.50 [64.75-113.50] | 141.94 (106.45) | 99.00 [74.00-168.00] | 2.29  (Welch) | .028 |
| Glucose | mg/dL | 86.80 (10.46) | 85.00 [81.00-92.00] | 99.55 (20.54) | 92.00 [85.00-116.00] | 3.45  (Welch) | .001 |
| Insulin | µIU/mL | 15.15 (14.14) | 9.00 [4.85-23.80] | 50.70 (63.39) | 23.50 [6.70-71.00] | 3.20  (Welch) | .003 |
| BUN | mg/dL | 11.97 (3.17) | 12.00 [9.00-14.00] | 13.18 (3.78) | 13.00 [11.00-15.00] | 1.89 | .061 |
| Creatinine | mg/dL | 0.77 (0.16) | 0.76 [0.64-0.89] | 0.77 (0.15) | 0.80 [0.66-0.87] | -0.06 | .952 |
| Cystatin C | mg/L | 0.87 (0.09) | 0.88 [0.82-0.92] | 0.89 (0.12) | 0.88 [0.80-0.95] | 0.50  (Welch) | .618 |

**Note.** Mean difference was calculated as higher-dysregulation profile minus control profile. Independent-samples t-tests assuming equal variances were used for mean comparisons when Levene's test was not significant; Welch's t-test was used when Levene's test indicated unequal variances. Because the profiles were derived from these biomarkers, inferential comparisons are provided to aid descriptive interpretation and should not be treated as independent confirmatory tests. Abbreviations: DHEA-S, dehydroepiandrosterone sulphate; hs-CRP, high-sensitivity C-reactive protein; IL-6, interleukin-6; TC, total cholesterol; HDL-C, high-density lipoprotein cholesterol; TG, triglycerides; BUN, blood urea nitrogen. Values are shown as mean (SD) and median [IQR].

**Supplementary Table S4.** Comparisons by sex for demographic and main study variables

| Variable | Females  (*n* = 84) | Males  (*n* = 81) | *t* | *p* |
| --- | --- | --- | --- | --- |
| Age, M (SD) | 22.61 (2.61) | 23.52 (2.51) | -2.29 | .023 |
| SPM, M (SD) | 52.10 (4.82) | 52.67 (5.67) | -0.70 | .486 |
| CD-RISC, M (SD) | 63.68 (12.38) | 66.68 (13.49) | -1.49 | .138 |
| BDI, M (SD) | 5.76 (5.38) | 4.60 (6.44) | 1.25 | .213 |
| GF:S, M (SD) | 9.23 (0.52) | 9.05 (0.76) | 1.75 | .082 |
| GF:R, M (SD) | 9.08 (0.56) | 9.04 (0.58) | 0.52 | .604 |

Abbreviations: SPM, Standard Progressive Matrices; CD-RISC, Connor-Davidson Resilience Scale; BDI, Beck Depression Inventory; GF:S, Global Functioning: Social; GF:R, Global Functioning: Role.

**Supplementary Figure S1.** Standardized biomarker profiles for alternative 3-, 4-, 5-, and 6-profile solutions.

Panels A–D correspond to the 3-, 4-, 5-, and 6-profile solutions, respectively. Biomarkers are organized by physiological domain. These alternative solutions are provided to aid interpretation of model enumeration and were not retained as the primary profile solution. Abbreviations: DHEA-S, dehydroepiandrosterone sulphate; hs-CRP, high-sensitivity C-reactive protein; IL-6, interleukin-6; TC, total cholesterol; HDL-C, high-density lipoprotein cholesterol; TG, triglycerides; BUN, blood urea nitrogen. Error bars indicate 95% confidence intervals for the group means of the biomarker z-scores.
